# Molecular simulation of the Kv7.4[ΔS269] mutant channel reveals that ion conduction in the cavity is perturbed due to hydrophobic gating

**DOI:** 10.1016/j.bbrep.2020.100879

**Published:** 2020-12-16

**Authors:** Md Harunur Rashid

**Affiliations:** a School of Engineering, RMIT University, Melbourne, Victoria, 3001, Australia; b Department of Mathematics and Physics, North South University, Bashundhara, Dhaka, 1229, Bangladesh

## Abstract

Mutations in the voltage-gated potassium channel Kv7.4 (encoded as KCNQ4) lead to the early onset of non-syndromic hearing loss, which is significant during language acquisition. The deletion of the S269 pore residue (genetic Δ mutation) in Kv7.4 has been reported to be associated with hearing loss. So far, there is no mechanistic understanding of how this mutation modulates channel function. To understand the role of S269 in ion conduction, we performed molecular dynamics simulations for both wild type and ΔS269 mutant channels. Simulations indicate that the ΔS269 mutation suppresses the fluctuations in the neighboring Y269 residue and thereby consolidates the ring formed by I307 and F310 residues in the adjacent S6 helixes in the cavity region. We show that the long side chains of I307 near the entrance to the cavity form a hydrophobic gate. Comparison of the free energy profiles of a cavity ion in Kv7.4 and Kv7.4[ΔS269] channels reveals a sizable energy barrier in the latter case, which suppresses ion conduction. Thus the simulation studies reveal that the hydrophobic gate resulting from the ΔS269 mutation appears to be responsible for sensorineural hearing loss.

## Highlights

• DeltaS269 mutation in the Kv7.4 channel is associated with hearing loss (SNHL).
• The mutation effects on channel function are studied via MD simulations.
• DeltaS269 mutation imposes a constriction at the cavity to suppress K^+^ conductance.
• Understanding the mutation effects on channel function will help to treat SNHL.

## 1 Introduction

Hearing impairment is a common communication disorder that is heterogeneous both genetically and clinically. It can be categorized as conductive and sensorineural hearing loss (SNHL). With surgical treatment, we can overcome conductive hearing loss but the treatment of hearing impairment is limited to hearing aids and cochlear implants. For the treatment of SNHL, pharmacotherapy is useful in limited cases [1]. A proper molecular-level understanding of SNHL could open up new avenues for developing novel therapeutics.

The degeneration of cochlea and cochlea's components are largely responsible for SNHL [1,2]. For sound transmission through the ear, the ion composition of the cochlear duct is critically important [3,4]. The fluid within the cochlear duct, endolymph, has a very high concentration of potassium (K^+^) ions. High cation concentration generates a net positive potential in the cochlear duct [5]. This positive potential is considered as one of the basic requirements of sound perception. It is well reported that several channels function to maintain a high level of K^+^ ions in endolymph.

Ion channels, and especially K^+^ channels, play an important role in the auditory system [6,7]. Among the various type of cochlear cells, hair cells, which convert sound stimuli to neural signals, have been a central therapeutic target for hearing loss.

Kv7.4 encoded by the KCNQ4 gene is a functional potassium channel in the hair cells. The dysfunction of this ion channel is responsible for sensorineural hearing loss from moderate to dominant deafness. Genetic mutations in KCNQ4 are mostly responsible for the nonsyndromic SNHL hearing loss with DFNX (X: X-linked) kinds. Research has been conducted on hearing loss associated with mutations [[8], [9], [10], [11], [12], [13], [14], [15], [16], [17]] and most of them are for nonsyndromic progressive sensorineural hearing loss. A recent report [18] (Hearing Research, 2020, 388, 107884) also mentioned few mutations associated with the sensorineural hearing loss but the SNHL hearing loss on frame-shift and deleted (/truncated mutations) residues are noted in a few cases [8,19,20].

Mechanical sound activates the Kv7.4 channels in the outer hair cells to transmit sound in the form of ion transfer from endolymph. To maintain K^+^ concentration in the endolymph, a recycling complex process takes place where Kv7.4 plays a role [6]. For a few mutation cases, it is reported that Kv7.4 openers can improve progressive hearing loss [21,22]. But channel modulators are ineffective in the case of pore mutations [21].

Here we perform a simulation study of Kv7.4[ΔS269] (genetic deletion of S269) to get insights into the structural changes and ion conduction in the channel compared to wildtype Kv7.4. We hope that insights gained on the effect of the ΔS269 mutation in Kv7.4 would lead us to novel treatments to address sensorineural hearing loss (SNHL).

Because Kv7.4 is involved in converting mechanical sound to neural signals, our finds will be useful in studying the effect of electromagnetic waves on lipid embedded Kv7.x channels.

## 2 Methods

**Homology Model.** Kv7.4 (encoded as KCNQ4) and Kv7.4[ΔS269] homology models were built from the structure of rKv1.2 (encoded as KCNA2; PDB ID: 2R9R). We created homology structures from the Swiss Model [23]. Using Modeller Program [24], we also constructed an asymmetric unit of residue deleted channel and found no significant shift in RMSD by aligning with the Swiss Model structure (Fig. S1). We note that constructing a homology model from the structure of Kv7.1 (PDB ID: 5VMS) has a drawback because it has 5 extra residues in the sequence alignment (Fig. S2 (a)). Nevertheless, the backbone alignment of the homology model with the Kv7.1 structure shows no significant differences (Figs. S2(b) and S2(c)), boosting confidence in the model structure.

**Simulation details.** *Trans*-membrane protein (Kv7.4/Kv7.4[ΔS269]) is embedded in a Phosphatidylcholine (POPC) lipid bilayer and solvated with explicit water molecules in a simulation box. We add only KCl to neutralize and keep buffer concentration to 150 mM. The NAMD code (version 2.10) is used for all MD runs [25]. The CHARMM22 [26] protein with CMAP [27] corrections, CHARMM36 [28] lipid, TIP3P water, and ion from CHARMM36 force field parameters are used for simulating the membrane proteins. The models are set for 50 ns MD equilibration and subsequent 100 ns production run, using the previously developed protocols [29].

**Potential of mean force (PMF) calculations.** PMF calculations are performed to examine the free energy profiles of K^+^ ion from the cytoplasm to the ion channel cavity. The PMFs are constructed by umbrella sampling on the cavity-ion path. The umbrella windows are generated at 0.5 Å steps by pulling the K^+^ ion from the cytoplasm to the cavity. At each window, the Z-coordinate of the K^+^ ion is sampled using a harmonic potential with a 10 kcal/mol/Å^2^ force constant. To construct the PMF profiles we used the WHAM code [30]. The final PMFs are calculated from the total production data where each umbrella sampling window runs for 3.5 ns MD simulations.

## 3 Results and discussion

**Fig. 1:**
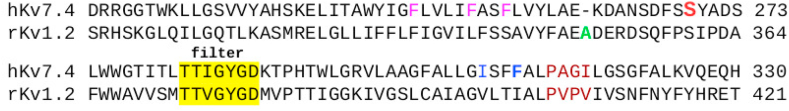
The pore domain sequence alignment of hKv7.4 and rKv1.2. Conserved PVPV motif in Kv1.x channels and its corresponding PAGI motif in Kv7.x are highlighted in brown color. The missing residue in the sequence alignment is shown in green color.

As the crystal structure of Kv7.4 is not available, we rely on the homology model for simulation studies. The homology model of Kv7.4 is created from the crystal structure of Kv1.2 (PDB: 2R9R) with the sequence alignment in Fig. 1. The alignment has ~ 30% similarity in sequence alignment that is desirable for the homology model [31]. There is a missing Ala residue in the sequence alignment (green residue in Fig. 1), which is deleted in the Kv7.4 model.

### 3.1 Tyr and Trp residues at the protein-lipid interface in the Kv7.4 potassium channel

**Fig. 2:**
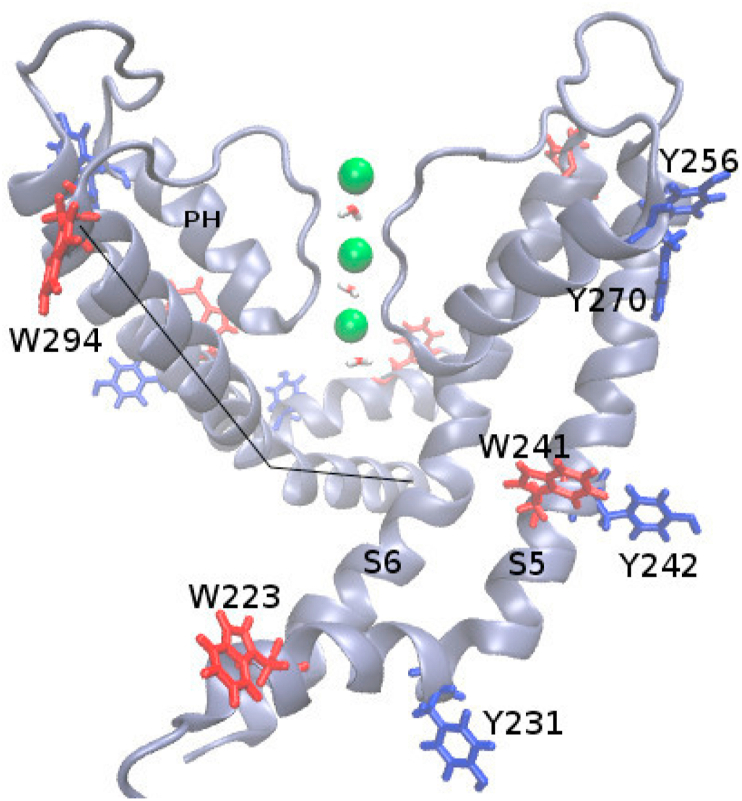
Lipid bound Tyr and Trp in Kv7.4 represented with licorice in blue and red colors, respectively in two cross monomers. Potassium ions in the filter are represented with green balls. Helices are labeled with S5, S6, and PH (pore helix). For clarity, lipids are not shown. The kink angle in S6 helix is defined with the intercepting of two lines at P314 in the PAGI motif (considering backbone nitrogen atoms along the line of W294 to P314 and P314 to V325). (For interpretation of the references to color in this figure legend, the reader is referred to the Web version of this article.)

Residues Y231, Y242, Y256, Y270, W223, and W294 are exposed to the lipid-protein interface (Fig. 2) and encircle the membrane protein within the lipid bilayer. Among these residues, Y270 belongs to the pore helix, W294 is in S6 helix, and the other residues are in S5 helix. The Tyr and Trp residues in the S5 helix of tetrameric Kv7.4 channel are outnumbered compared to other Kv channels (as Kv1.X and KcsA). There are three Phe residues (pink codes in Fig. 1) in S5 helix that are also bound to the lipid bilayer. Overall these hydrophobic residues make S5 helices well attached to the bilayer, compared to other potassium channels. Thereby Y270 residues in protein-lipid interface act as floats [32] and provide stability to the pore helices.

### 3.2 Structural changes due to the deletion of S269

**Fig. 3:**
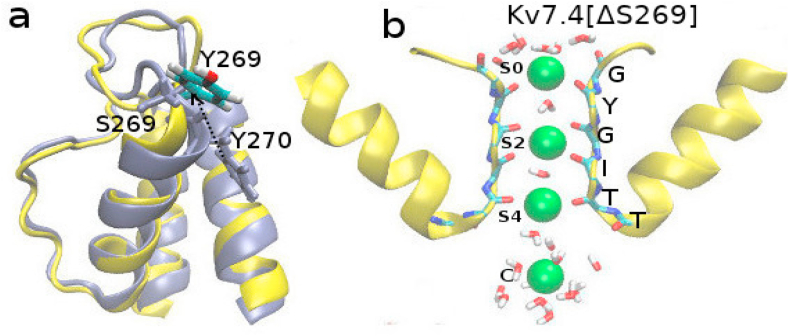
(a)The aligned structure of Kv7.4 (ice blue) and Kv7.4[ΔS269] (yellow) in one monomer. The positions of S269 and Y270 are explicitly shown in Kv7.4 with ice-blue and Y269 of Kv7.4[ΔS269] shown with atom colors. (b) Filter and cavity ion positions are indicated with Si (i runs from 0 to 4) and C respectively. (For interpretation of the references to color in this figure legend, the reader is referred to the Web version of this article.)

**Fig. 4:**
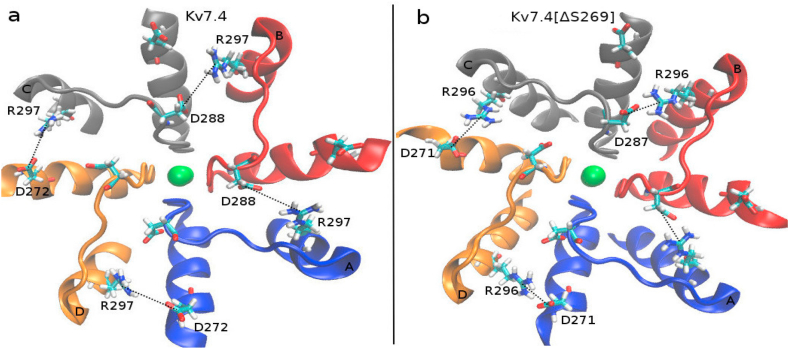
The Arg-Asp interactions (indicated by dashed lines). (a) Interactions between R297-D272 and R297-D288 in the WT channel. (b) Interactions between R296-D271 and R296-D287 in the mutant channel.

The wild-type and ΔS269 channels are aligned to see the structural changes in the pore region (Fig. 3a). The deletion of S269 reduces the length of the pore helix and relocates Y269 closer to the position of S269 in WT Kv7.4 (Fig. 3a). As a consequence, Y269 becomes freer to fluctuate between lipid headgroups and bulk water, losing its previous role as a float. To examine this change, we plot the chi1 torsional angle distribution of the Y270 residue in WT and Y269 residue in the mutated channel. There is a clear shift in the chi1 distributions between the WT and mutant channels (Fig. S3). The pore helices also become more flexible and rearrange to form new interhelical salt bridges between the Arg and Asp residues as shown in Fig. 4.

To show the formation of the salt bridges quantitatively, we compare the Arg(N)-Asp(O) distances between the WT and mutant Kv7.4 channels (Table 1). It is seen that the *N*-O distances in Kv7.4[ΔS269] contract relative to the WT distances and become smaller than 3 Å in all cases, which signals that salt bridges are formed between the Arg and Asp residues as indicated in Fig. 4.

**Table 1:** The average values of R297(N)-D272(O) and R297(N)-D288(O) distances in Kv7.4, and R296(N)-D271(O) and R296(N)-D287(O) distances in Kv7.4[ΔS269] channels from the last 100 ns production run.

	R297(N)-D272(O) (Å)	**R296(N)-D271(O) (Å)**
	Kv7.4	Kv7.4 [ΔS269]
**C-D**	3.2 ± 0.3	2.6 ± 0.2
**D-A**	5.6 ± 0.2	2.7 ± 0.2
	R297(N)-D288(O) (Å)	R296(N)-D287(O) (Å)
	**Kv7.4**	**Kv7.4[ΔS269]**
**A-B**	6.3 ± 0.4	2.9 ± 0.3
**B-C**	5.5 ± 0.3	2.6 ± 0.2

The formation of the interchain Arg-Asp salt bridges in the outer pore has influence far beyond and affects the I-F (Ile-Phe) ring region, which will be discussed below.

### 3.3 Energetics of the cavity ion (C) from PMFs calculation

**Fig. 5:**
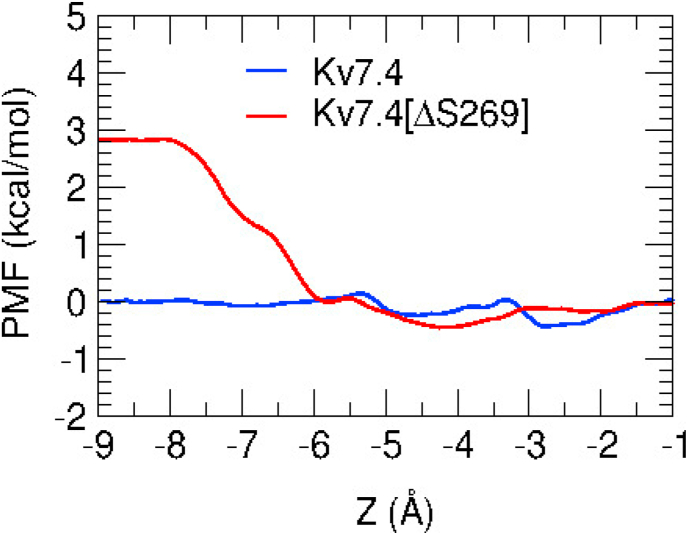
PMFs of a K^+^ ion between cavity and cytoplasm, where the ion is moved from the cytoplasmic site, at Z < - 9 Å toward cavity. . In WT Kv7.4 (blue), the PMF is almost flat but it exhibits a substantial barrier in the [ΔS269] mutant (red). The convergence of the PMFs are shown in Fig. S4 and in Fig. S5. (For interpretation of the references to color in this figure legend, the reader is referred to the Web version of this article.)

In the initial configuration, water molecules (W) and potassium ions occupy the selectivity filter in a sequence of W-S1-W-S3-W (where Si (i = 0, 1, 2, 3, 4) corresponds to the K^+^ binding sites in the selectivity filter), which is used in constructing the Kv7.4 and Kv7.4[ΔS269] simulation models [33,34]. One K^+^ ion is also placed in the cavity in the conduction-waiting state [35]. The created model state in the filter transforms to S0-W-S2-W-S4 state during early equilibration of the WT Kv7.4 channel. Interestingly, in the mutant channel, we have observed a new state, S0-W-S2-W-S4-C, where a fourth K^+^ ion remains in the cavity (Fig. 3b). In WT Kv channels, when the S4 position is occupied by an ion, it is less likely to have a K^+^ ion in the cavity due to the strong electrostatic repulsion [36]. But this could be possible if structural changes take place in the cavity region due to the deletion of S269. We will first try to understand whether such a state is energetically possible in Kv1.4[ΔS269] or not. To this end, we calculate the PMFs for a cavity ion in the WT (blue PMF) and the mutant Kv7.4 channels (red PMF) (Fig. 5).

In the WT Kv7.4 channel, the PMF profile in the cavity is rather flat, exhibiting no energy barriers for a K^+^ ion in the cavity to exit to the cytoplasm. In contrast, in the Kv7.4[ΔS269] channel, an energy barrier appears at z=−6 Å, which rises to about 3 kcal/mol at z = −8 Å. This barrier (3 kcal/mol ~ 5 kT) is sufficient to suppress ion conduction from cavity to cytoplasm even in the presence of a repulsive force from an ion at S4 position (Z = 4.8 Å). The formation of this new ionic state, i.e. S0-W-S2-W-S4-C, helps to explain how the ΔS269 mutation causes dysfunction of the Kv7.4 channel. The likely cause of this energy barrier is the conformational changes in the I-F ring region, where the ring of I308 residues form a hydrophobic gate [35]. We discuss the gating in the next section.

### 3.4 I-F ring and hydrophobicity

**Fig. 6:**
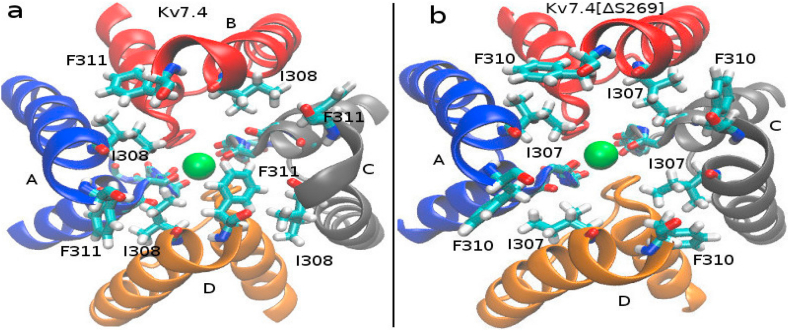
Hydrophobic interaction between the Ile and Phe residues form a ring structure around the S6 helixes. (a) Ring structure in wild type channel. (b) Ring structure in the S269 deleted channel. The snapshots are taken from the cytoplasmic side, for both channels. Four monomers are indicated with A, B, C, and D. Ion in the filter is shown in green. (For interpretation of the references to color in this figure legend, the reader is referred to the Web version of this article.)

The I308 and F311 residues on S6 helices from adjacent monomers in Kv7.4 and the corresponding I307 and F310 residues in Kv7.4[ΔS269] form a ring structure in the respective channels (Fig. 6). The formation of this ring facilitates the long side chains of the Ile residues to remain directed towards the position of the S4 ion in the filter. Conformational changes in this ring in the mutant channel may be responsible for the energy barrier in Fig. 5. To this end, we calculate C_α_−C_α_ distances between the F and I residues of adjacent monomers (Fig. 6) for the WT and mutant channels (Table 2).

**Table 2:** C_α_−C_α_ distances of Ile and Phe of adjacent monomers in the I-F ring.

Kv7.4	C_α_−C_α_ (Å)	Kv7.4[ΔS269]	C_α_−C_α_ (Å)
**1308(A)- F311(B)**	8.1 ± 0.6	1307(A)- F310(B)	7.7 ± 0.6
**1308(B)- F311(C)**	8.6 ± 0.7	1307(B)- F310(C)	7.6 ± 0.5
**1308(C)- F311(D)**	7.7 ± 0.7	1307(C)- F310(D)	7.8 ± 0.7
**1308(D)- F311(A)**	8.3 ± 0.9	1307(D)- F310(A)	7.8 ± 0.8

**Fig. 7:**
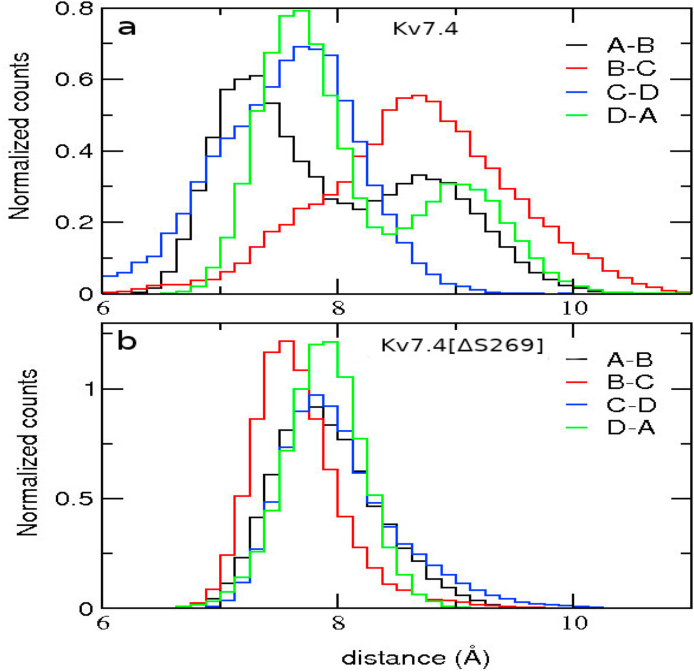
Distributions of the C_α_- C_α_ distances of Ile-Phe residues in Fig. 6. (a) I308-F311 C_α_- C_α_ distance distributions in Kv7.4 (b) I307-F310 C_α_- C_α_ distance distributions in Kv7.4[ΔS269]. The distribution between adjacent monomers represented with A-B (black), B-C (red), C-D (blue), and D-A (green) in WT and mutant channels. (For interpretation of the references to color in this figure legend, the reader is referred to the Web version of this article.)

The C_α_−C_α_ distances decrease by ~ 0.5 Å on average in Kv7.4[ΔS269] over WT Kv7.4. To get a better insight, we also plot the distributions of C_α_−C_α_ distances (Fig. 7). In contrast to WT Kv7.4 where the distributions are quite different, they become very similar in Kv7.4[ΔS269] indicating a more orderly symmetric structure in the mutant channel. This symmetric and less fluctuating I-F distribution indicates a uniform hydrophobic interaction in the ring residues over the WT channel. For further clarification, we also plot the chi2 torsional angle distributions for the Phe and Ile ring residues (Fig. S6), which also show the symmetric distributions in Kv7.4[ΔS269], over WT Kv7.4.

To quantify the effect of the ΔS269 mutation on the cavity structure further, we calculate the average cross monomer C_α_−C_α_ distances between the I308 side chains in WT Kv7.4 and the corresponding I307 side chains in Kv7.4[ΔS269]. The distances are reduced by 2.7 Å for A-C and 3.1 Å for B-D cross monomers in the Kv7.4[ΔS269] channel compared to that of the WT channel (Table 3). Shorter cross monomer distances between the Ile residues indicate a narrowing of the pore region. The smaller cavity holds a substantially smaller number of water molecules (water molecules within 8 Å of the Ile residue), which drop from about 50 in WT to 30 in the mutant channel.

**Table 3:** Ile-Ile cross monomer C_α_−C_α_ distances (in Å).

Kv7.4	distance	Kv7.4[ΔS269]	distance
**I308(A)- I308(C)**	14.1 ± 0.5	I307(A)- I307(C)	11.3 ± 0.4
**I308(B)- I308(D)**	14.2 ± 0.5	I307(B)- I307(D)	11.1 ± 0.3
			

Narrowing of the pore could create a steric barrier for the passage of K^+^ ions in this hydrophobic gate region. To examine this possibility, we calculate the average cross monomer C_δ_-C_δ_ distances for the Ile residues in Table 3, which give an indication of the space available for K^+^ ions. The distances are reduced by 1.5 Å for A-C and 2.2 Å for B-D cross monomers in the Kv7.4[ΔS269] channel compared to that of the WT channel (Table 4). Because this region is hydrophobic, a K^+^ ion has to pass with its hydration shell intact, otherwise, there will be an energetic penalty due to perturbation of the hydration shell. The radius of a hydrated K^+^ ion is 3.5 Å. Including the van der Waals radius of C_δ_ atoms (1.7 Å), we estimate that the minimal C_δ_-C_δ_ distance for passage of a hydrated K^+^ ion is about 10.4 Å. Comparing this value with the C_δ_-C_δ_ distances in Table 4, it is seen that the gate opening is sufficiently large in WT Kv7.4 but not in Kv7.4[ΔS269]. Thus the energy barrier in the mutant PMF (Fig. 5) arises from the suboptimal opening of the hydrophobic gate in Kv7.4[ΔS269].

**Table 4:** Ile-Ile cross monomer C_δ_-C_δ_ distances (in Å).

Kv7.4	distance	Kv7.4[ΔS269]	distance
I308(A)- I308(C)	10.8 ± 0.5	I307(A)- I307(C)	9.3 ± 0.5
I308(B)- I308(D)	10.9 ± 0.4	I307(B)- I307(D)	8.7 ± 0.5

## 4 Conclusion

A number of mutations in Kv7.4 are responsible for sensorineural hearing loss (SNHL). Molecular-level studies for deletion mutations in the pore region are very limited. Such mutations usually modulate channel functions. Here we have explored the effect of the ΔS269 mutation on ion permeation. We have shown that this mutation releases the Y270 side chains and weakens the stability of the pore helices, which form new interhelical salt bridges and ultimately transform the open state channel to a semi-open state. As this state is the consequence of a constricted I-F ring structure and not a functional waiting state, it will change the rate of ion conduction in Kv7.4[ΔS269]. Because Kv7.4 is involved in K^+^ recycle process in the inner ear, changes in the ion conduction rate will lead K^+^ imbalance in the endolymph, which is believed to be the potential cause of sensorineural hearing loss on pore residue mutations. This aspect needs further experimental study. This study also provides valuable molecular information about Kv7.4, such as the presence of I-F ring, the hydrophobicity in the cavity region, and the key hydrophobic residues in the protein-lipid interface, which differentiate it from other Kv channels.

Because channel openers are not useful for pore residue mutations in Kv7.x, novel small molecule drugs must be developed to treat SNHL. They need to bind in between the S269 position and surrounding lipids (drug target) to work as a float. This could help to stabilize the pore helices and thus prevent their rearrangement which leads to a semi-open channel.

## Authorship statement

Manuscript Title: Molecular simulation of the Kv7.4[ΔS269] mutant channel reveals that ion conduction in the cavity is perturbed due to hydrophobic gating.

Authorship contributions: The research work is single-authored and hence, works reported in the manuscript credited to the corresponding author. Non-author contributions are highlighted in the Acknowledgements section of the manuscript.

## Declaration of competing interest

The author declares no conflict of interest.
