# Supplementary material for: Molecular simulation of the Kv7.4[ΔS269] mutant channel reveals that ion conduction in the cavity is perturbed due to hydrophobic gating
Source: Biochem Biophys Rep. 2020 Dec 16;25:100879. doi: 10.1016/j.bbrep.2020.100879 (PMC7749434; doi:10.1016/j.bbrep.2020.100879)
Supplement: Multimedia component 1 [file mmc1.docx]

Supplementary Information

Molecular simulation of the Kv7.4[ΔS269] mutant channel reveals that ion conduction in the cavity is perturbed due to hydrophobic gating

Md Harunur Rashid^1, 2^

^1^School of Engineering, RMIT University, Melbourne, Victoria, 3001, Australia.

^2^Department of Mathematics and Physics, North South University, Bashundhara, Dhaka, 1229, Bangladesh


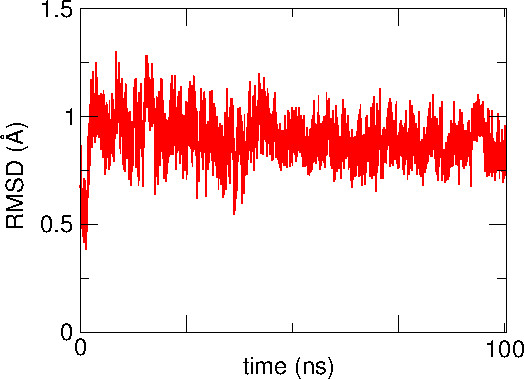


Fig. S1: RMSD of backbone heavy atoms for one asymmetric unit.


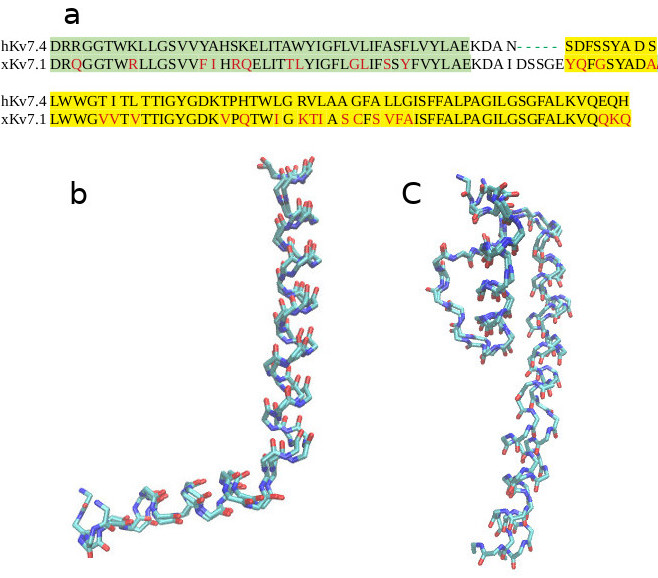


Fig. S2: (a) The pore domain sequence alignment of hKv7.4 and xKv7.1(from the frog). The ‘5’ missing residues in the sequence alignment is shown with green dashes and mismatched residues are shown with red colour. (b) Backbone alignment of the S5 helixes highlighted with green in the sequence alignment. (c) Backbone alignment of the S6 and PH helixes, highlighted with yellow colour in the sequence alignment.


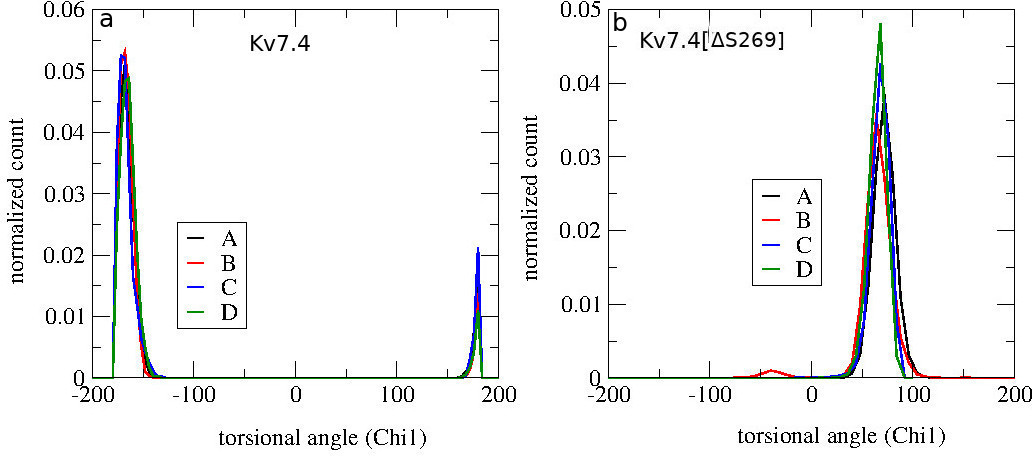


Fig. S3: Torsional angle chi1 distribution (a) chi1 distribution of Y270 residue in Kv7.4 (b) Chi1 distribution of Y269 residue in Kv7.4[ΔS269].


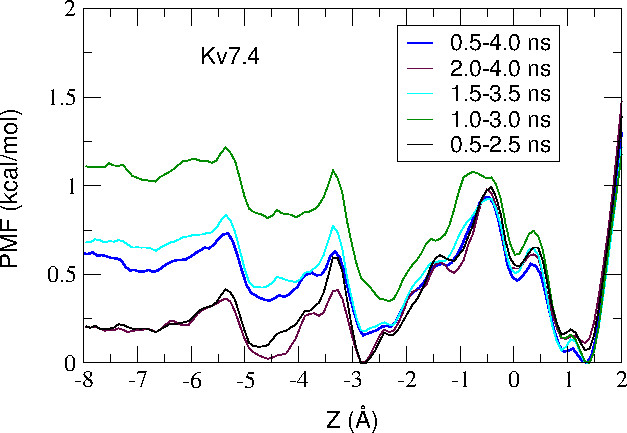


Fig. S4: Convergence of PMFs for 2 ns block data in Kv7.4. 2 ns blocks are prepared with 0.5 ns shifting data. The PMFs are fluctuating from 0.2 kcal/mol to 1.2 kcal/mol. Therefore, we calculate the final PMF from the last 3.5 ns data and average fluctuation is calculated from this value. 0.5 ns is discarded for early equilibration.


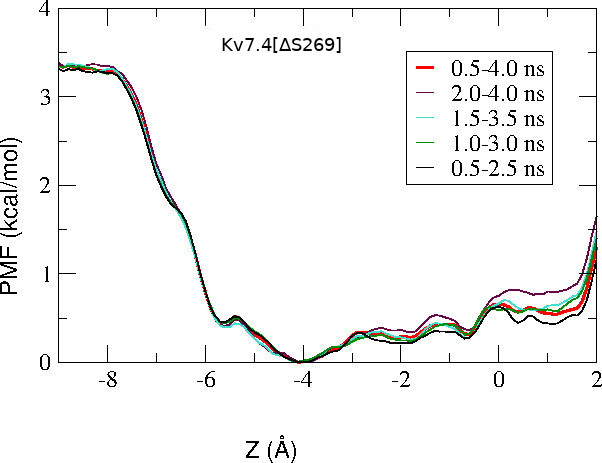


Fig. S5: Convergence of PMFs for 2 ns block data in Kv7.4[ΔS269]. 2 ns blocks are prepared with 0.5 ns data shifting. The final PMF is calculated from the last 3.5 ns data and average fluctuation is calculated from this value. 0.5 ns is discarded for early equilibration.


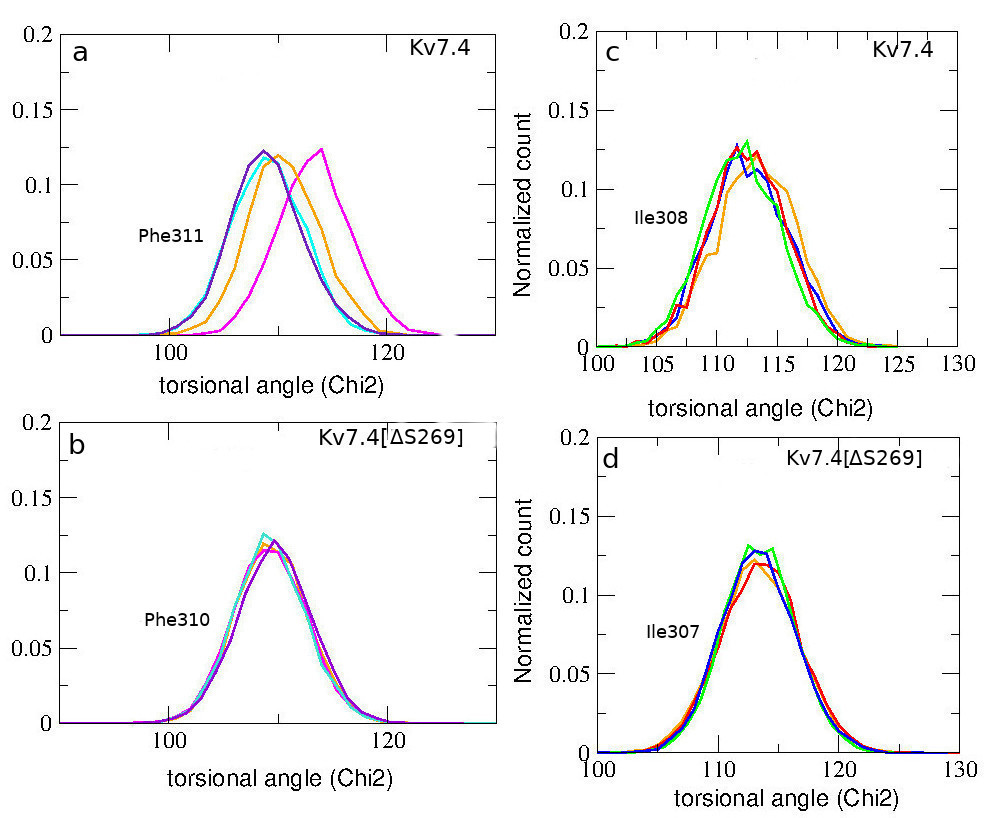


Fig. S6: Torsional angle Chi2 distribution (a) Chi2 distribution of Phe311 residue in Kv7.4 (b) Chi2 distribution of Phe310 residue in Kv7.4[ΔS269]. (c) Chi2 distribution of Ile308 residue in Kv7.4 (d) Chi2 distribution of Ile307 residue in Kv7.4[ΔS269]. Four different colours in the figures represent the distribution of the torsional angles in four monomers.
